# The Role of Computed Tomography Perfusion in Various Focal Liver Lesions

**DOI:** 10.7759/cureus.32420

**Published:** 2022-12-11

**Authors:** Vignesh Gadupudi, Rajoo Ramachandran, Venkata Sai Pulivadula Mohanarangam

**Affiliations:** 1 Department of Radiology, Sri Ramachandra Institute of Higher Education and Research, Chennai, IND

**Keywords:** hepatic metastasis, hepatic abscess, hepatic cyst, hepatocellular carcinoma (hcc), liver, perfusion ct

## Abstract

Background

This study aims to identify the potential advantages of quantitative determination of various focal liver pathologies, identify lesion hemodynamics, and distinguish benign and malignant pathologies based on CT perfusion (CTP) parameters.

Methodology

In this study, we examined 36 patients using contrast-enhanced CT (CECT) and proposed inclusion and exclusion criteria. Of the 36 patients, 18 had malignant lesions and 14 had benign lesions. CTP was performed on patients comprising cases of hepatocellular carcinoma (HCC), metastasis, hemangiomas, hepatic cysts, and hepatic abscess. Images were post-processed and analyzed to calculate various perfusion parameters such as blood flow (BF), blood volume (BV), permeability surface (PS), mean transit time (MTT), the hepatic arterial fraction (HAF), and induced residue fraction time of onset (IRFTO). Parameters were compared between benign and malignant lesions, and descriptive analysis was performed for individual lesions.

Results

Data were analyzed with IBM SPSS Statistics (IBM Corp., Armonk, NY, USA). IRFTO showed the area of the curve (AOC) = 0.659, *P*-value = 0.040, sensitivity 66.7%, and specificity 64.3%. BV showed AOC = 0.659, *P*-value = 0.040, with a cutoff value of 1.26, sensitivity of 66.7%, and specificity of 64.3%. BF showed AOC = 0.786 and *P-*value = 0.006, with a cutoff value of 171.2, sensitivity of 83.3%, and specificity of 78.6%. MTT showed AOC = 0.778 and *P*-value = 0.008, with a cutoff value of 6.94, sensitivity of 77.8%, and specificity of 78.6%. Statistically significant changes were observed in the perfusion parameters in the BV, BF, MTT, and IRFTO.

Conclusions

The noninvasive CT liver perfusion technique makes it possible to compare the hemodynamic changes in healthy and sick liver tissues, identify focal liver lesions, and evaluate the effectiveness of tumor therapy.

## Introduction

A minimally invasive technique, called perfusion CT, allows for an extremely accurate assessment of tissue perfusion. Modern multidetector CT scanners are ideal for measuring perfusion because of their great spatial and temporal resolution [1].

The advent of multidetector CT has given rise to the acquisition of images with higher quality and accuracy. In addition to assessing recipients' vascular structure, it is frequently used to preoperatively select living, related liver donors. This imaging method is also used for the initial assessment and follow-up of the majority of patients with hepatic metastases, and it provides important details on the existence and severity of extrahepatic illness as well as the number, size, and distribution of hepatic metastases [1].

The main aim of this study is to identify the potential advantages of quantitative determination of various focal liver pathologies, identify the lesion hemodynamics, and distinguish benign and malignant pathologies based on the CT perfusion (CTP) parameters.

The objectives of this study are to identify focal liver lesions hemodynamics by performing CT dynamic contrast perfusion, know the accuracy of CT liver perfusion in the characterization of focal liver lesions to aid better radiological interpretation, and provide optimal treatment strategy and post-process the acquired images to facilitate the analyzation of liver function through measurement of various tissue perfusion parameters such as the mean transit time (MTT), portal liver perfusion (PLP), blood flow (BF), blood volume (BV), hepatic arterial perfusion index (HPI), and alkaline phosphatase (ALP).

## Materials and methods

The ethics committee approval and informed consent were obtained from all patients before this study. This is a period study from 2019 to 2021, with a sample size of 36. All patients with incidental detection of liver lesions or those who have been referred to our institute for liver pathology/planned for liver transplantation were included in our study. Patients with contrast allergy, pregnant patients, or patients with elevated renal profile parameters were excluded.

The images were transferred to the CT workstation, and post-processing was performed. Liver perfusion parameters such as BF, BV, MTT, PLP, ALP, and HPI were calculated. CTP parameters were obtained, details of liver diseases were tabulated, statistical analysis was done, and perfusion parameters were correlated with disease severity findings depicted on CT.

CT liver perfusion protocol

All studies were performed using GE Revolution EVO (128 -slice CT, GE Healthcare, Chicago, IL, USA). In our study, the patient was prepared for nothing by mouth (NPO) 4 hours before the examination. RFT values were thoroughly checked, and an explanation of the procedure was given.

**Figure 1:**
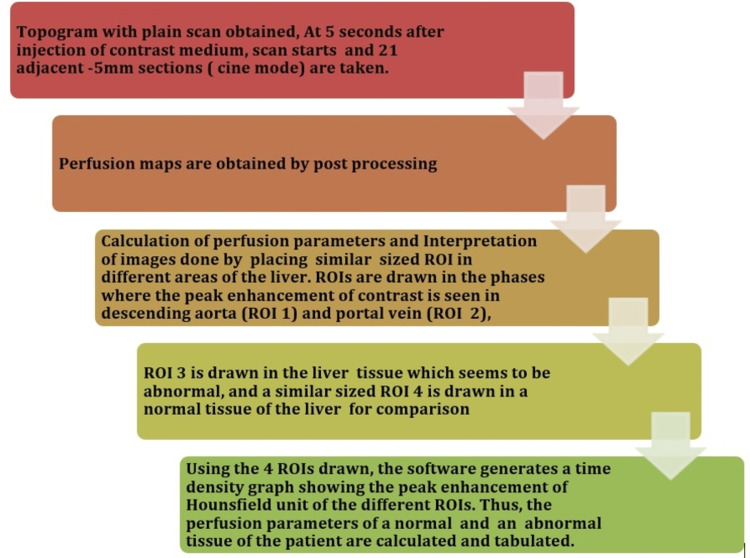
CTP protocol. Figure credits: Vignesh Gadupudi CT, computed tomography, CTP, CT perfusion; ROI, region of interest

The patient was positioned on the CT scanner in a supine position with feet-first orientation, the landmark was positioned at the xiphisternum, the standardized *CT liver perfusion *protocol (Figure 1) was selected, and a series of high-temporal resolution images were acquired following rapid intravenous administration of iodinated nonionic contrast material (Iohexol 350 mg/mL) using pressure injector with the flow rate of 4-4.5 mL/second and dose of 1.2 mL/kg body weight.

## Results

The comparison of BV to predict the malignancy using the receiver operating characteristic (ROC) curve, which shows the area of the curve as 0.813, *P*-value = 0.003 < 0.05, with a 95% confidence interval of 0.663 to 0.963, which is statistically significant with the cutoff value of 14.98, sensitivity of 77.8%, and specificity of 78.6%, is shown in Table 1. 

**Table 1:** The area under the curve for the variable BV. BV, blood volume

Area	Standard error	Asymptotic sig. (P-value)	Asymptotic (95% confidence interval)	Cutoff	Sensitivity	Specificity
0.813	0.077	0.003	0.663-0.963	14.98	77.8%	78.6%

The comparison of BF to predict the malignancy using the ROC curve, which shows the area of the curve as 0.786, *P*-value = 0.006 < 0.05, with a 95% confidence interval of 0.571 to 1, which is statistically significant with a cutoff value of 171.2, sensitivity of 83.3%, and specificity of 78.6%, is shown in Table 2.

**Table 2:** The area under the curve for the variable BF. BF, blood flow

Area	Standard error	Asymptotic sig. (P-value)	Asymptotic (95% confidence interval)	Cutoff	Sensitivity	Specificity
0.786	0.110	0.006	0.571-1	171.20	83.3%	78.6%

The comparison of MTT to predict the malignancy using the ROC curve, which shows the area of the curve as 0.778, *P-*value = 0.008 < 0.05, with 95% confidence interval of 0.564 to 0.992, which is statistically significant with the cutoff value of 6.94, sensitivity of 77.8%, and specificity of 78.6%, is shown in Table 3. 

**Table 3:** The area under the curve for the variable MTT. MTT, mean transit time

Area	Standard error	Asymptotic sig. (P-value)	Asymptotic (95% confidence interval)	Cutoff	Sensitivity	Specificity
0.778	0.109	0.008	0.564-0.992	6.94	77.8%	78.6%

The comparison of IRFTO to predict the malignancy using the ROC curve, which shows the area of the curve as 0.659, *P*-value = 0.040 < 0.05, with a 95% confidence interval of 0.534 to 0.895, which is statistically significant with the cutoff value of 1.26, sensitivity of 66.7%, and specificity of 64.3%, is shown in Table 4. 

**Table 4:** The area under the curve for the variable IRFTO. IRFTO, induced residue fraction time of onset

Area	Standard error	Asymptotic sig. (P-value)	Asymptotic (95% confidence interval)	Cutoff	Sensitivity	Specificity
0.714	0.092	0.040	0.534-0.895	1.26	66.7%	64.3%

Representative cases

Case 1: Simple Hepatic Cyst

**Figure 2:**
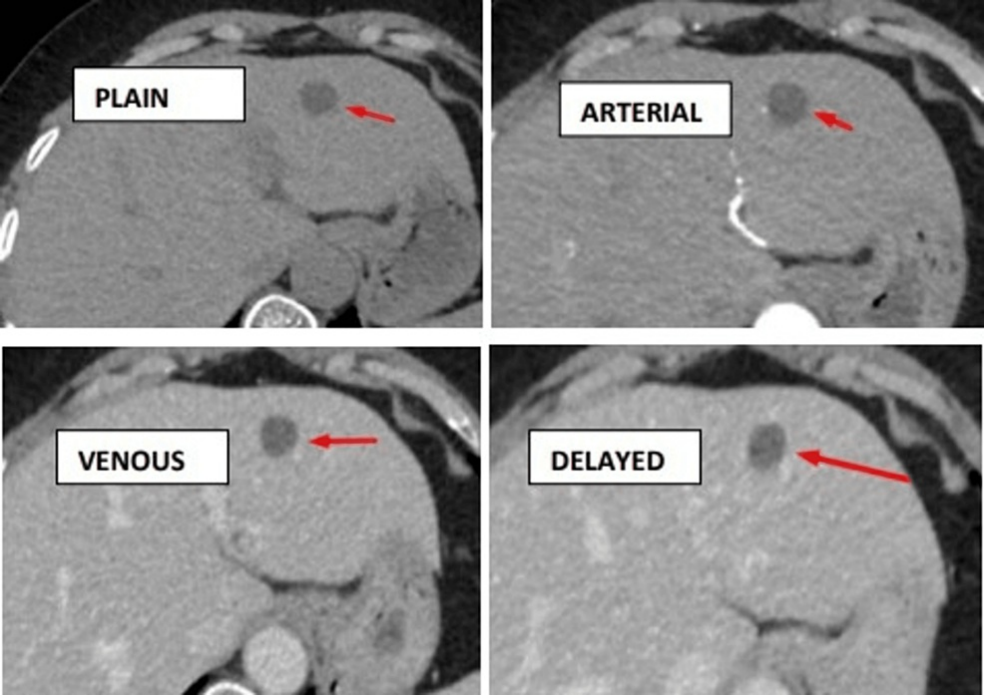
Plain study showing hypodense lesions in segment III of the liver, with no enhancement in the arterial, venous, and delayed phases - the hepatic cyst.

Plain study showing hypodense lesions in segment III of the liver, with no post-contrast enhancement in the arterial, venous, and delayed phases (Figure 2).

**Figure 3:**
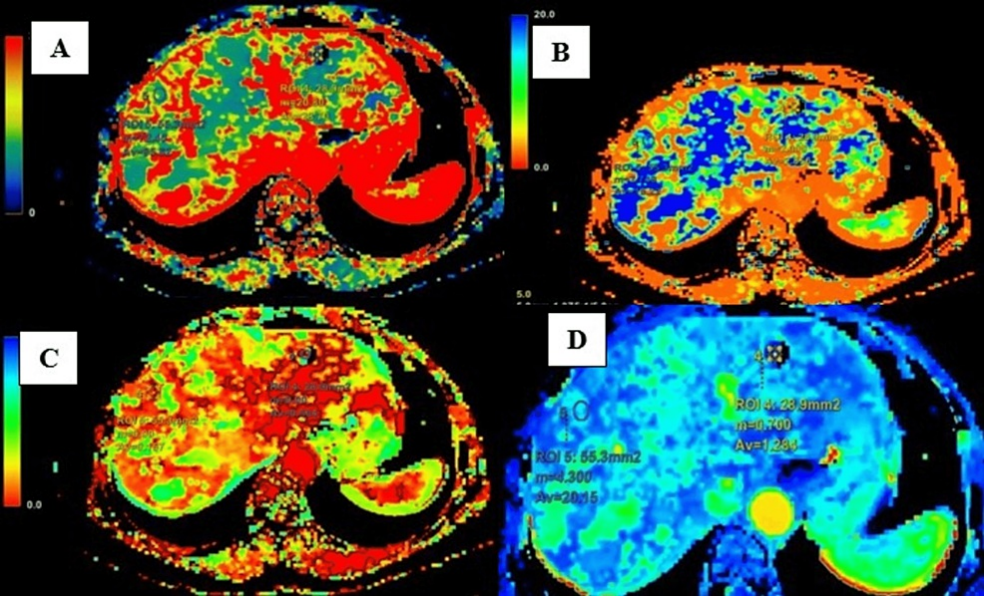
Maps for (A) BF, (B) MTT, (C) IRFTO, and (D) BV. BF, blood flow; MTT, mean transit time; IRFTO, induced residue fraction time of onset; BV, blood volume

CT perfusion maps for variables BF, MTT, IRFTO, and BV were obtained (Figure 3). 

Perfusion parameters in the lesion area and normal liver parenchyma in the case of the hepatic cyst are shown in Table 5. The parameters in hepatic cysts showed reduced intralesional BV, BF, PS, and MTT with raised IRFTO.

**Table 5:** The perfusion parameters in the lesion area and normal liver parenchyma in the case of hepatic cysts. BF, blood flow; MTT, mean transit time; IRFTO, induced residue fraction time of onset; BV, blood volume; PS, permeability surface; HAF, hepatic arterial fraction; T max, time to max; HEP ART, hepatic arterial

Parameter	Lesion	Normal
Average	16.83	91.1
Base	14.61	82.9
Time to peak	22.08	30.27
Positive enhancing integral	0.0212	0.0775
Mean slope of increase	0.74	1.002
BV	1.284	20.15
BF	28.7	85.82
MTT	2.856	15.22
IRFTO	0.564	0.157
T max	1.077	6.284
HAF	0.531	0
PS	3.215	72.54
HEP ART flow	15.03	0

Case 2: Flash Hemangioma

**Figure 4:**
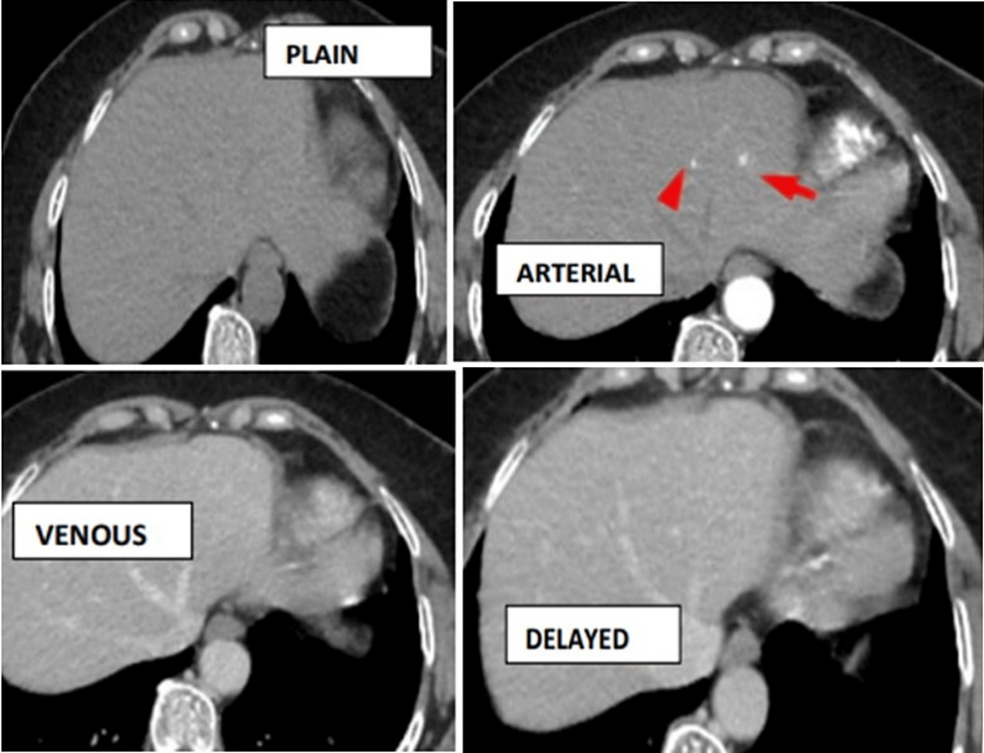
Plain study showing isodense lesions in segment III of the liver and intense homogenous arterial enhancement (arrow and arrowhead) with isodense lesions in the venous and delayed phases - flash-filling hemangioma.

Plain study showing isodense lesions in segment III of the liver. Post-contrast administration, the lesions show intense homogenous arterial enhancement (arrow and arrowhead) with washout in venous and delayed phases (Figure 4).

**Figure 5:**
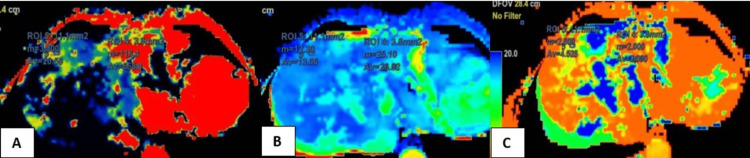
Maps for (A) BF, (B) BV, and (C) MTT. BF, blood flow; MTT, mean transit time; BV, blood volume

 CT perfusion maps for variables BF, BV, and MTT were obtained (Figure 5). 

Perfusion parameters in the lesion area and normal liver parenchyma in the case of hemangioma are shown in Table 6. The perfusion values in hemangioma showed increased intralesional BV, BF, and IRFTO, with a relatively reduced MTT.

**Table 6:** The perfusion parameters in the lesion area and normal liver parenchyma in the case of hemangioma. BF, blood flow; MTT, mean transit time; IRFTO, induced residue fraction time of onset; BV, blood volume; PS, permeability surface; HAF, hepatic arterial fraction; T max, time to max; HEP ART, hepatic arterial

Parameter	Lesion	Normal
Average	147	94.23
Base	97.12	87.91
Time to peak	19.67	273
Positive enhancing integral	0.462	0.0568
Mean slope of increase	6.935	0.789
BV	26.82	13.06
BF	765. 9	215. 7
MTT	2	4.528
IRFTO	3.368	1.502
T max	4.295	8.113
HAF	0.874	0.148
PS	88.3	5.717
HEP ART flow	668. 1	20.58

Case 3: Hepatocellular Carcinoma (HCC)

**Figure 6:**
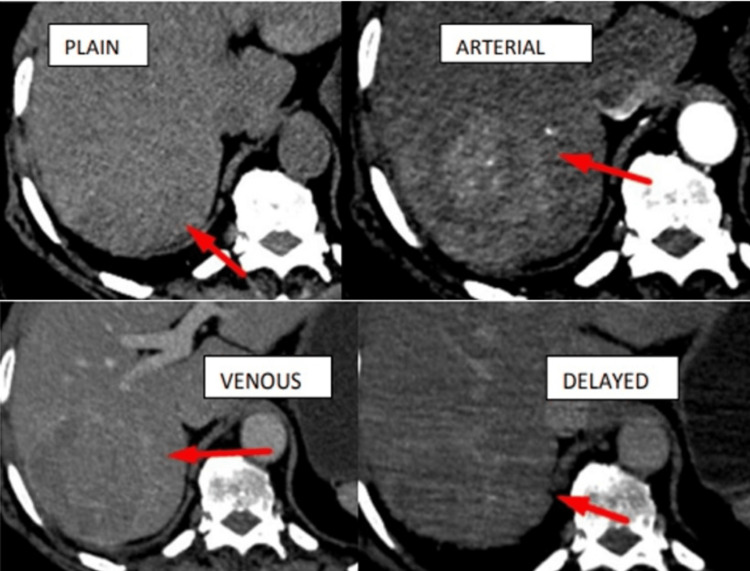
Plain study showing hypodense lesions in segment VIII of the liver and intense arterial enhancement with washout in the venous and delayed phases with cirrhotic background - HCC. HCC, hepatocellular carcinoma

Plain study showing a hypodense lesion in segment VIII of the liver, intense arterial enhancement with washout in the venous and delayed phases with a cirrhotic background (Figure 6).

**Figure 7:**
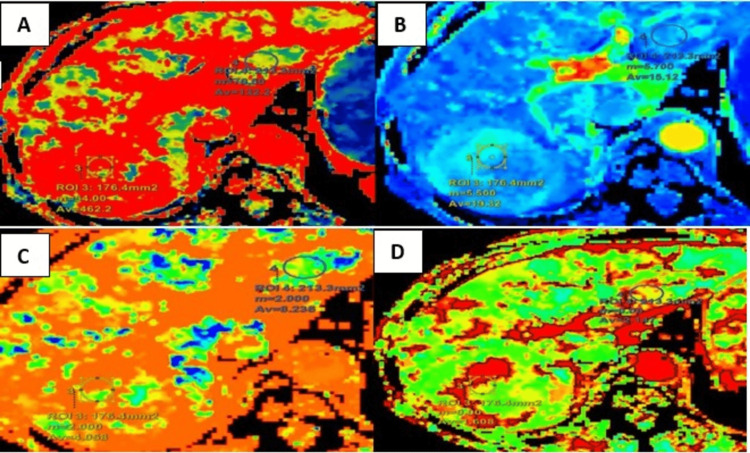
Maps for (A) BF, (B) BV, (C) MTT, and (D) PS. BF, blood flow; MTT, mean transit time; BV, blood volume; PS, permeability surface

 CT perfusion maps for variables BF, BV, MTT, and PS were obtained (Figure 7). 

Perfusion parameters in the lesion area and normal liver parenchyma in the case of HCC are shown in Table 7. The perfusion values in HCC showed increased intralesional BV and BF with a relatively reduced PS, MTT, and IRFTO.

**Table 7:** The perfusion parameters in the lesion area and normal liver parenchyma in the case of hepatocellular carcinoma. BF, blood flow; MTT, mean transit time; IRFTO, induced residue fraction time of onset; BV, blood volume; PS, permeability surface; HAF, hepatic arterial fraction; T max, time to max; HEP ART, hepatic arterial

Parameter	Lesion	Normal
Average	97.87	78.61
Base	81.81	72.44
Time to peak	23.11	28.95
Positive enhancing integral	0.137	0.0536
Mean slope of increase	2.009	0.8
BV	19.32	15.12
BF	462. 2	132. 2
MTT	4.058	8.236
IRFTO	1.608	2.147
T max	3.172	6.13
HAF	0.342	0.0746
PS	12.28	22.45
HEP ART flow	81.45	11.12

Case 4: Hepatic Abscess

**Figure 8:**
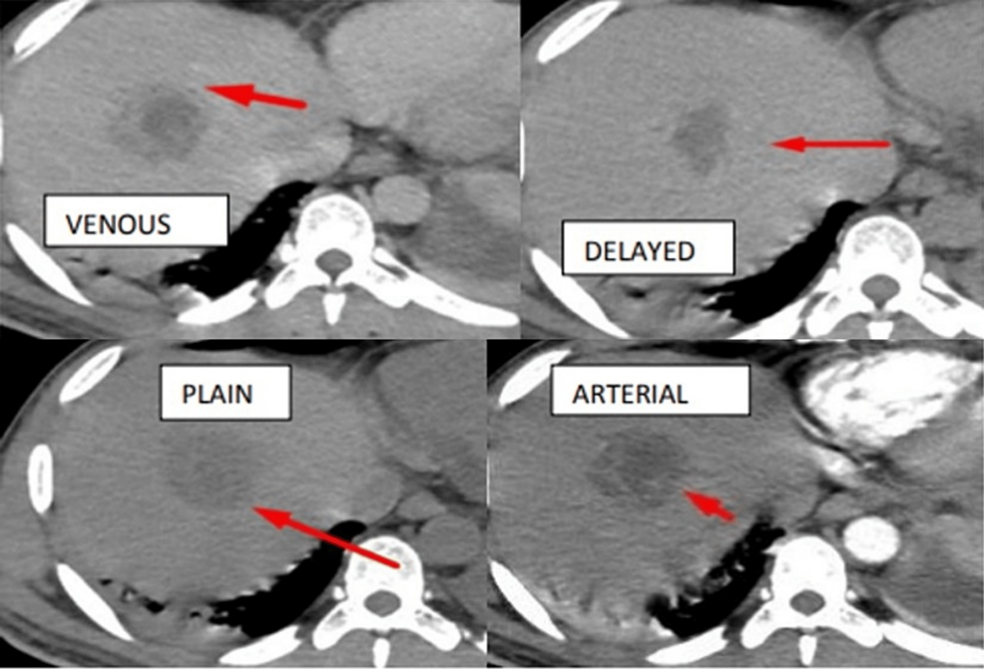
Plain study showing hypodense lesions in segment VII of the liver and central low-attenuation lesions (fluid-filled) surrounded by a high-attenuation inner rim and low-attenuation outer ring in the venous and delayed phases - the hepatic abscess.

Plain study showing hypodense lesions in segment VII of the liver and central low-attenuation lesions (fluid-filled) surrounded by a high-attenuation inner rim and low-attenuation outer ring in the venous and delayed phases (Figure 8).

**Figure 9:**
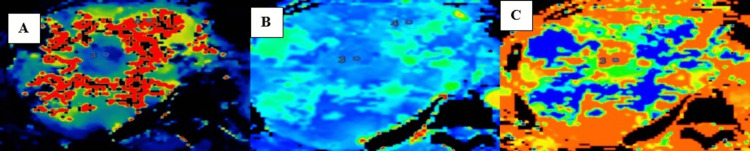
Maps for (A) BF, (B) BV, and (C) MTT. BF, blood flow; MTT, mean transit time; BV, blood volume

CT perfusion maps for variables BF, BV, and MTT were obtained (Figure 9).

Perfusion parameters in the lesion area and normal liver parenchyma in the case of hepatic abscess are shown in Table 8. The parameters in the hepatic abscess showed reduced intralesional BV, BF, and PS with raised MTT and IRFTO.

**Table 8:** The perfusion parameters in the lesion area and normal liver parenchyma in the case of hepatic abscess. BF, blood flow; MTT, mean transit time; IRFTO, induced residue fraction time of onset; BV, blood volume; PS, permeability surface; HAF, hepatic arterial fraction; T max, time to max; HEP ART, hepatic arterial

Parameter	Lesion	Normal
Average	33.32	69.51
Base	14.44	55.38
Time to peak	25.09	31.17
Positive enhancing integral	0.261	0.185
Mean slope of increase	0.987	1.987
BV	2	16.82
BF	11.14	88.26
MTT	33.4	12.27
IRFTO	3.938	1.382
T max	6.938	7.51
HAF	0.997	0.362
PS	33.84	168
HEP ART flow	28.3	32.26

Case 5: Metastasis

**Figure 10:**
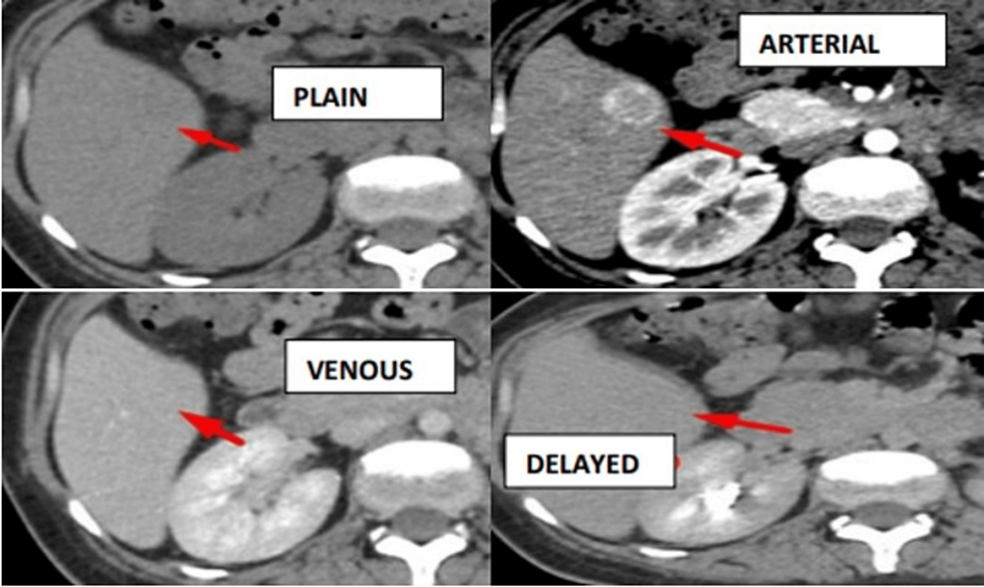
Plain study showing hypodense lesions in segment V of the liver and intense arterial enhancement with washout in the venous and delayed phases - metastasis.

Plain study showing hypodense lesions in segment V of the liver and intense arterial enhancement with washout in the venous and delayed phases - metastasis (Figure 10).

**Figure 11:**
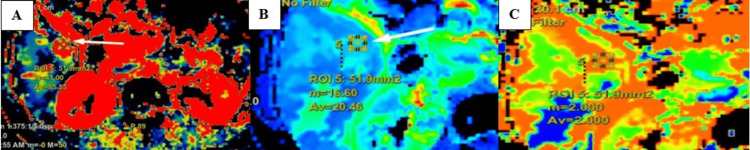
Maps for (A) BF, (B) BV, and (C) MTT. BF, blood flow; MTT, mean transit time; BV, blood volume

CT perfusion maps for variables BF, BV, and MTT were obtained (Figure 11).

Perfusion parameters in the lesion area and normal liver parenchyma in the case of metastasis are shown in Table 9. The perfusion values in metastasis showed increased intralesional BV, BF, and IRFTO with a relatively reduced MTT.

**Table 9:** The perfusion parameters in the lesion area and normal liver parenchyma in the case of metastasis. BF, blood flow; MTT, mean transit time; IRFTO, induced residue fraction time of onset; BV, blood volume; PS, permeability surface; HAF, hepatic arterial fraction; T max, time to max; HEP ART, hepatic arterial

Parameter	Lesion	Normal
Average	106. 2	99.36
Base	75.32	76.82
Time to peak	20.35	31.35
Positive enhancing integral	0.423	0.305
Mean slope of increase	2.514	1.476
BV	48.5	27.75
BF	613. 7	125. 6
MTT	8.456	18.67
IRFTO	0.627	0.198
T max	1.624	7.737
HAF	0.144	0.369
PS	63.03	35.5
HEP ART flow	85.55	32.61

## Discussion

Developmental, neoplastic, inflammatory, and other types of liver lesions are categorized as space-occupying liver lesions. Due to the importance of accurate liver imaging for effective therapy, it is necessary to discriminate between distinct liver lesions and develop individualized treatment plans for each.

Contrast-enhanced CT (CECT) is often insufficient to characterize focal liver lesions due to the variable enhancement pattern of liver lesions compared to the relatively constant enhancement pattern of normal liver parenchyma.

Assessing the hepatic and portal blood flow to the liver with CTP has a lot of potential. Liver pathologies alter the hemodynamics of the hepatic artery, hepatic vein, and portal vein due to their dual blood supply. Normal liver perfusion can be expressed as a ratio of blood supply from the hepatic artery and portal vein.

Perfusion parameters such as HAP, portal venous perfusion (PVP), HPI, BF, BV, MTT, IRFTO, and PS provide a quantitative determination of the lesion hemodynamics.

The liver perfusion is measured by HAP using arterial blood rather than portal blood. An increased vessel count would result in comparable increases in BF and BV. MTT is a reflection of perfusion pressure, in that a higher perfusion pressure forces blood to travel more quickly, which shortens the MTT [1].

PS is the product of permeability and total capillary endothelium surface area in a unit mass of tissue, expressed as mL/min/100 gm. PS represents the flux of solutes from blood plasma to the interstitial space and serves as a surrogate marker of the vascular leak [1].

Of the 36 patients, 72.2% were male and 27.8% were female, aged between 21 and 87 years, with a mean age of 58.6 years. Our study comprises maximum HCC, followed by metastasis and hepatic cysts, hepatic abscess, three cases of hemangioma, and two cases each of hepatic adenoma and fatty infiltration.

Perfusion parameters in hepatic cysts showed reduced intralesional BV, BF, PS, and MTT with raised IRFTO. Perfusion values in hemangioma showed increased intralesional BV, BF, and IRFTO with a relatively reduced MTT. The perfusion values in HCC showed increased intralesional BV and BF with a relatively reduced PS, MTT, and IRFTO.

Perfusion parameters in the hepatic abscess showed reduced intralesional BV, BF, and PS with raised MTT and IRFTO. Of all the CTP parameters assessed, four parameters, i.e., BF, BV, MTT, and IRFTO, showed statistical significance in differentiating benign and malignant lesions. Test variable BF had a cutoff of 171.2, with a sensitivity of 83.3 and specificity of 78.6 (*P-*value = 0.003). The test variable BV had a cutoff of 14.98, with a sensitivity of 77.8 and specificity of 78.6 (*P-*value = 0.006). The test variable MTT had a cutoff of 6.94, with a sensitivity of 77.8 and specificity of 78.6 (*P-*value = 0.008). The test variable IRFTO had a cutoff of 1.26 with a sensitivity of 66.7 and specificity of 64.3 (*P-*value = 0.040).

Using proposed inclusion and exclusion criteria, we examined 36 patients in this study using CECT. Of the 36 patients, 18 had malignant lesions and 14 had benign lesions.

Because the growth and migration of cancerous cells depend on the proliferation of new blood vessels through the process of tumor angiogenesis, tissue perfusion is crucial in oncology. Angiogenesis can be quantified to assess tumor growth at an early stage and produce prognostic, predictive, and surrogate power [1].

A quantitative framework for evaluating the vascular heterogeneity brought on by tumor angiogenesis is provided by CTP in metastases. Additionally, it makes it possible to evaluate how chemotherapy and radiation therapy affect tumor vascularity and perfusion [1].

CTP values in metastases showed increased intralesional BV, BF, and IRFTO with a relatively reduced MTT as compared to those of normal liver parenchyma. Increased BF and shorter MTT observed in this study correlate with the findings of Guyennon et al. [2]. These findings are similar to the findings in the studies by Miles et al. [3], Blomley et al. [4], Leggett et al. [5], and Reiner et al. [6] that reported a significantly increased HAP and decreased PVP in patients with liver metastases when compared with adjacent normal parenchyma.

PS of the metastatic lesion is less than the PS of normal liver parenchyma in this study. However, the PS of the lesion and healthy liver tissue differ depending on the target organ [4].

Total BF and BV are substantially higher in metastases than in HCC, even though BF and BV can rise in both metastasis and HCC when compared with the surrounding normal parenchyma. New blood vessel development is crucial for growth and tumor spread. The distribution of perfusion parameters will change as a result of the enhanced tumor perfusion brought on by the increasing microvascular density [5].

Increased HAP and HPI in HCC specifically reflect the growth of new unpaired arterial blood vessels and a blood supply most exclusively derived from arterial circulation in HCC nodules. These perfusion results correlate with the studies by Ippolito et al. [7], Sahani et al. [8], Zhu et al. [9], and Marquez et al. [10] that demonstrated increased BV, BF, HAP, and HPI in HCC. Furthermore, in HCC-associated cirrhosis, increased HAP and decreased PVP found in this study could be due to the fact stated by van Beers et al. [11] that there is more artery overflow in cirrhosis and less important portal flow.

The main added advantage of this study when compared to the available literature is that we could establish a cutoff for a few CTP parameters such as BF, BV, MTT, and IRFTO to differentiate benign versus malignant liver lesions. Our study is the only study to have performed descriptive analysis and established mean values of various perfusion parameters for focal liver lesions.

The limitation of this study is the smaller sample size. Analysis of the variables and CTP parameters would have been better with a higher sample size. Another limitation of this study is the smaller size of the hemangioma. Larger lesions help in the accurate placement of the region of interest (ROI) in the enhancement part, and accordingly, CTP parameters alter.

No one has yet established a cutoff to distinguish between benign and malignant liver lesions using the CTP parameters. This study is unique as it includes both benign and malignant focal liver lesions, and we were able to statistically demonstrate the sensitivity and specificity of the perfusion parameters to aid in the differentiation of lesions.

In conclusion, CTP gives morphological data, functional knowledge of the tissue microenvironment, and quantitative evaluation of the lesion hemodynamics differentiating the malignant and benign processes.

## Conclusions

A noninvasive method, called CT liver perfusion, provides a quantitative evaluation of hemodynamic changes in healthy and sick liver tissues, which would otherwise be challenging to detect with standard CT. A promising method for identifying primary or metastatic cancers is liver CTP, which offers functional information on the microcirculation of healthy parenchyma and can be utilized to distinguish focal lesions of the liver. CTP liver imaging in an oncology setting aids in evaluating the effectiveness of systemic or local tumor therapy, predicting early response to anticancer medicines, and monitoring tumor recurrence after therapy.
